# Through-Hole, Self-Ordered Nanoporous Oxide Layers on Titanium, Niobium and Titanium–Niobium Alloys in Aqueous and Organic Nitrate Electrolytes

**DOI:** 10.1002/open.201100012

**Published:** 2012-02-10

**Authors:** Robin Kirchgeorg, Wei Wei, Kiyoung Lee, Seugli So, Patrik Schmuki

**Affiliations:** [a] Department of Material Science WW4-LKO, University of Erlangen-NurembergMartensstraße 7, 91058 Erlangen (Germany), Fax: (+49) 9131-852-7582 E-mail: schmuki@ww.uni-erlangen.de

**Keywords:** nanotubes, niobium pentoxide, self-organization, through-hole morphology, titanium dioxide

In the past decades, a variety of self-aligned functional oxides have successfully been grown on a wide range of metals using electrochemical, self-organizing anodization. The earliest reports on highly ordered oxide structures included porous aluminum oxide layers grown by optimized anodization of Al in oxalic acid.^1^ These nanoporous oxides found a considerable number of direct applications, such as size-exclusion filters^2^ and waveguide structures,^3^ or sacrificial uses, such as templates for secondary material deposition for the production of nanowires and tubes.^4^, ^5^

A very versatile self-organizing anodization approach was introduced in 1999 by Zwilling et al., which used fluoride-containing electrolytes for the fabrication of ordered TiO_2_ nanotube arrays on Ti.^6^ These fluoride-based electrolytes were optimized over the last ten years to enable the growth of self-organized oxide layers on many metals and alloys, including Ti,^7^ Zr,^8^, ^9^ Hf,^10^ Nb,^11^, ^12^ Ta,^13^ W,^14^ Ti–W,^15^ and Ti–Nb.^16^ A detailed overview can be found in Ref. 7.

In 2005, Masuda et al., followed by others,^16^–^19^ showed that by using perchlorate or chloride electrolytes, another type of nanotubes, the so-called rapid-breakdown anodization (RBA) nanotubes could be grown on Ti and W surfaces. This process was later extended to Ti–Nb, Ti–Zr and Ti–Ta alloys to form mixed-oxide nanostructures.^20^, ^21^ In this anodization approach, the formation of tubes occurs with a high current flow, and tubes grow as bundles from a specific surface site on the metal into the electrolyte.^17^–^21^ Due to the localized nature of the process and high current densities, mechanistically, the formation process was attributed to repeated anodic breakdown events of the surface oxide layer.^18^

Over the past ten years, considerable efforts have been directed toward the finding of other electrolyte types that would lead to the formation of self-organized nanostructured metal oxides. While nitrate-based electrolytes were used to etch Ti through a porous alumina template and, thus, can form etch channels,^22^ we recently showed that nitrate-based electrolytes also may be a promising new route to achieve truly self-organized oxide structures in the context of Ti and Ta anodization.^23^ In the present work, we explore the use of nitrate-containing electrolytes for the formation of self-organized (template-free) oxide structures on Ti, Nb and Ti–Nb alloys.

Ordered TiO_2_-based nanoscale structures are particularly interesting in terms of applications in catalysis,^24^ solar cells,^25^ photolysis,^26^ sensing,^27^ and electrochromic devices.^16^ Nb is an important element in combination with Ti, since composite oxides can be formed, or TiO_2_ can be Nb-doped for an alteration of the electronic properties.^28^ For TiO_2_ nanotubes, it has been shown that in large concentrations the incorporation of Nb leads to lattice widening^16^ and is, therefore, beneficial in ion insertion devices (e.g., electrochromic applications and ion intercalation batteries). In smaller concentrations, Nb acts as a donor species to enhance the performance of TiO_2_-based solar cells and water splitting reactions.^28^–^30^ Herein, we demonstrate that anodization in nitrate-based electrolytes can be tuned to form ordered, nanoporous oxide structures, not only on Ti, but also on Nb and Ti–Nb alloys. Moreover, in contrast to any other previously reported electrolyte types, this nitrate-based anodization leads directly to a through-hole morphology for all investigated structures, i.e., where the pores are open at the top and bottom.

A series of preliminary anodization experiments for all the metals in various aqueous and ethylene glycol-based nitrate electrolytes were carried out, screening parameters being concentration, pH, and water content. The results showed that on Ti, Nb and Ti45–Nb, ordered porous layers could be grown (Figure 1). In aqueous electrolytes, a sufficiently high anodization voltage had to be applied to initiate the growth of a porous layer with an aligned pore structure. For the three investigated materials, the conditions to achieve a defined layer growth are different in each case. For Ti, well-ordered pores could be observed for anodization in HNO_3_. The example shown in Figure 1 a resulted in an oxide-layer thickness of approximately 10 μm. The inset pictures in Figure 1 show that regular pore channels with a diameter of 10–20 nm and a through-hole morphology could be obtained. Using Nb, a stable compact oxide film, instead of a porous oxide layer, was formed in all explored aqueous nitric acid electrolytes. However, when anodization was carried out in an organic nitrate electrolyte, well-defined porous layers could be grown. Figure 1 b shows the cross section of a self-organized nanoporous Nb oxide layer formed in an ammonium nitrate electrolyte. The resulting layer thickness was approximately 4 μm, and the pore diameter of the through-hole morphology was approximately 10–15 nm.

**Figure 1:**
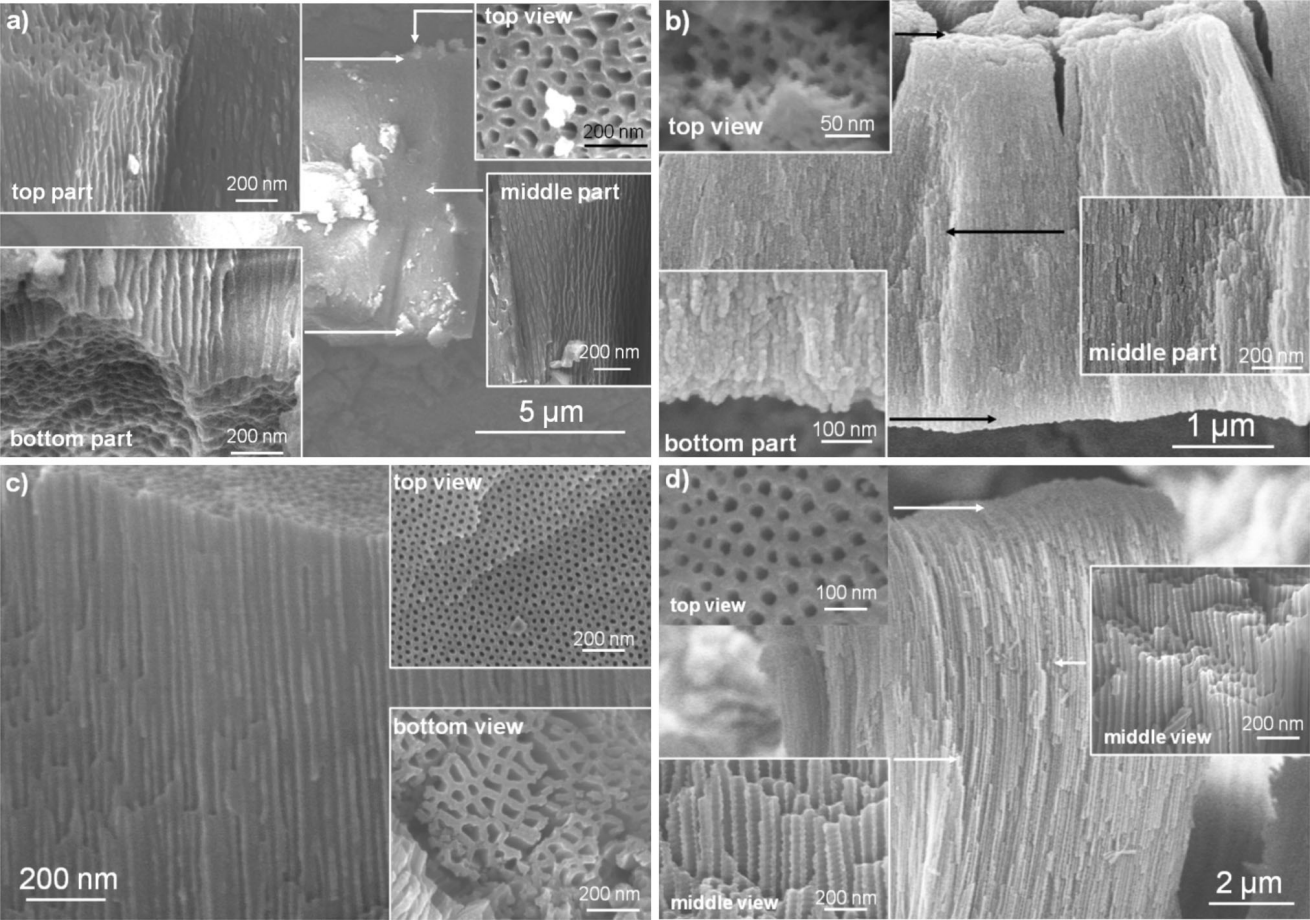
SEM images of cross sections. a) Nanoporous TiO_2_ with a through-hole morphology prepared in 0.1 m HNO_3_ at 40 V for 180 s. The insets show the top, middle and bottom part of the cross section and the top view. b) Nanoporous Nb_2_O_5_ prepared in 0.2 m NH_4_NO_3_ and 3 % (*v*/*v*) H_2_O in ethylene glycol at 40 V for 300 s. The insets show the middle and bottom part of the cross section and the top view. c) Nanoporous Ti45–Nb alloy with through-hole morphology prepared in 0.1 m HNO_3_ at 40 V for 180 s. The insets show the top and the bottom view. d) The Ti45–Nb alloy with well-aligned pores anodized in 0.2 m NH_4_NO_3_ and 3 % (*v*/*v*) H_2_O in ethylene glycol for 1800 s. The insets show the top and the middle part of the layer.

A defined and self-organized pore morphology was obtained for the Ti45–Nb alloy in both types of electrolytes—aqueous and organic nitrate. Examples of the highly aligned pore channels and the through-hole morphology for aqueous and organic electrolytes are shown in Figure 1 c and d. Whereas a pore diameter of 10–20 nm, comparable to Ti, was found in the aqueous electrolyte, a pore diameter of 20–50 nm was measured in the organic electrolyte. Compared to pure Ti, a thinner layer of approximately 4 μm was obtained with the alloy in the aqueous electrolyte, which indicates that the alloyed Nb reduces the oxide growth rate. The oxide layer thickness (∼10 μm) grown in the organic electrolyte under the same conditions, on the other hand, is comparable to pure Nb (Figure 1 b and d).

Figure 2 shows current density–time curves observed during anodization, illustrating the different anodizing behaviors of the various substrates in aqueous and organic electrolytes. In general, the current density in the organic electrolyte is lower than in the aqueous electrolyte by an order of magnitude. In the aqueous electrolyte, very high current densities are directly observed with a gradual decay for Ti and the Ti–Nb alloy. Ordered porous layers were formed with both substrates with the thickness increasing with time. This type of behavior was seen for applied voltages in the range of 15–40 V. However, when anodization was carried out in ethylene glycol electrolytes, much higher currents were observed, and self-organized porous oxide layers could be grown in the voltage range of 15–60 V. In the organic electrolyte, the alloy showed an increase in current up to 10 mA cm^−2^, followed by a decay. A similar trend was observed in the aqueous electrolyte, where highly self-organized nanoporous Ti–Nb oxide layers could also be observed in a potential range of 20–40 V. Overall, regarding the electrochemical conditions under which ordered, porous layers were formed, it may be deduce that in NO_3_^−^-based electrolytes and under the investigated conditions, a mechanism lying between the high-field case,^7^, ^31^–^33^ which is responsible for self-ordered nanoporous structures on Al in oxalic acid or fluoride-induced self-ordered structures, and the breakdown mechanism, which forms RBA tubes,^15^ is responsible for order and oxide growth.

**Figure 2:**
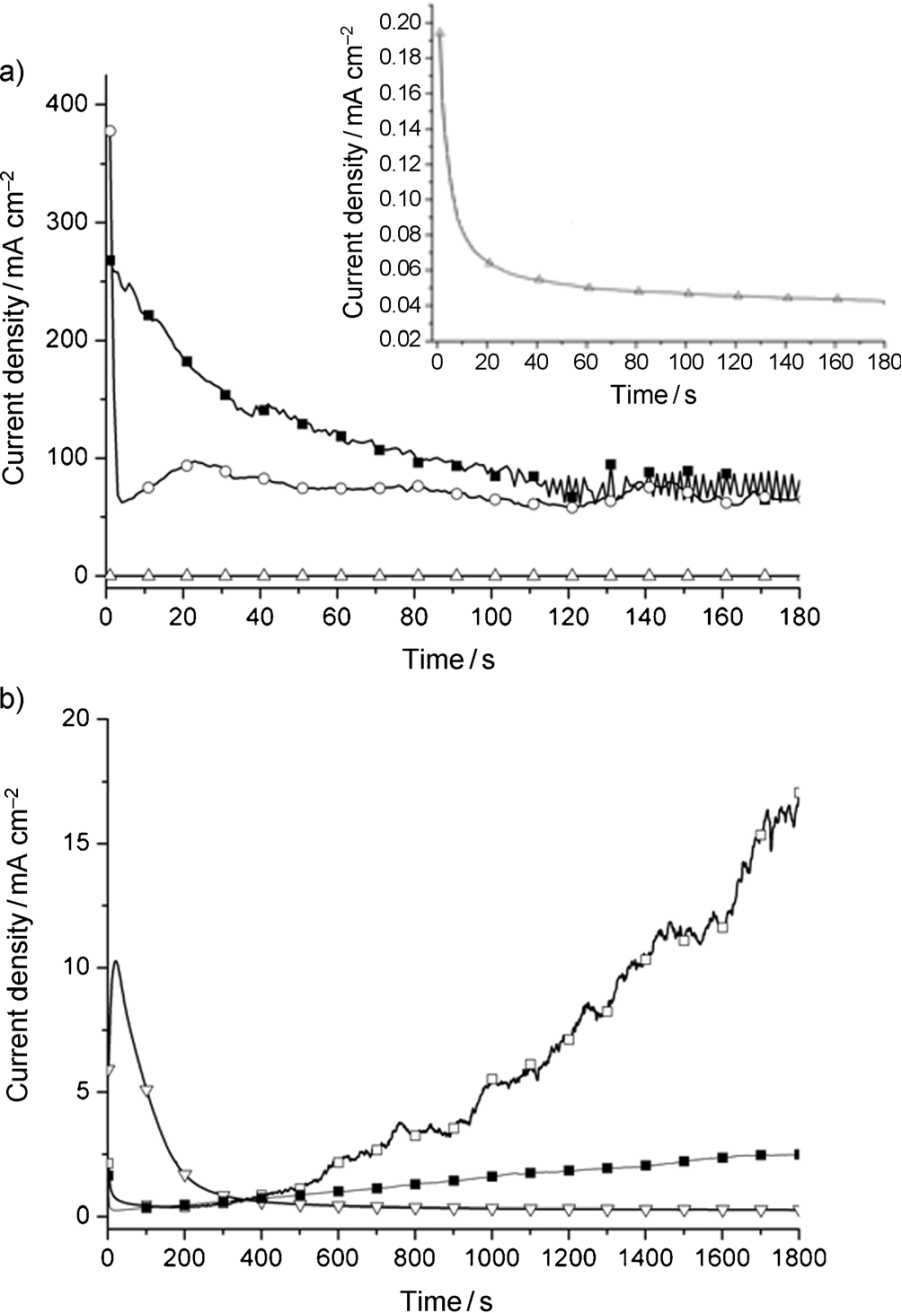
Current transient of a) Ti (▪), Nb (▵) and the Ti45–Nb alloy (○) in aqueous 0.1 m HNO_3_ at 40 V for 180 s, and b) Nb at 30 (▪) and 40 V (□) and the Ti–Nb alloy at 40 V (▿) in 0.2 m NH_4_NO_3_ and 3 % (*v*/*v*) H_2_O in ethylene glycol.

To obtain information on the structure and composition of the oxide layers, X-ray diffraction (XRD) and energy dispersive X-ray (EDX) measurements were carried out. The XRD patterns in Figure 3 a–c show the as-formed nanoporous oxide layers on Ti, Nb and the Ti–Nb alloy to be amorphous. EDX measurements confirmed the as-formed layer on Ti to correspond to TiO_2_, and the layer on Nb to correspond to Nb_2_O_5_. The structures could be crystallized by an appropriate annealing treatment. Crystallization was carried out at 450 °C in air for TiO_2_ and at 550 °C in an N_2_ atmosphere for Nb_2_O_5_.^34^, ^36^ As seen from the XRD measurements, after annealing, the nanoporous oxide layers were present as anatase TiO_2_ with small amounts of rutile TiO_2_ in the case of Ti,^34^ and as monoclinic Nb_2_O_5_ for pure Nb.^35^ Upon annealing, the layer on the alloy showed conversion to a composite oxide (Figure 3 c), that is, individual anatase TiO_2_ and monoclinic Nb_2_O_5_ phases could be detected. EDX measurements of the annealed layer grown on the Ti45–Nb alloy in the aqueous or organic electrolyte yielded a composition of approximately 14 % Ti, 33 % Nb, 48 % O, 5 % C and 0 % N in both cases (Figure 4 a and b). The results are in line with the formation of a TiO_2_/Nb_2_O_5_ layer on top of the Ti–Nb substrate. It must be mentioned that the substrate is clearly contributing to the EDX signal. From this result, it becomes apparent that in the oxide structures, Nb_2_O_5_ is enriched compared with TiO_2_. This is in line with the electrochemical behavior observed in Figure 2 and literature reports that state TiO_2_ to be much more prone to dissolution in NO_3_^−^-based electrolytes than Nb_2_O_5_.^30^

**Figure 3:**
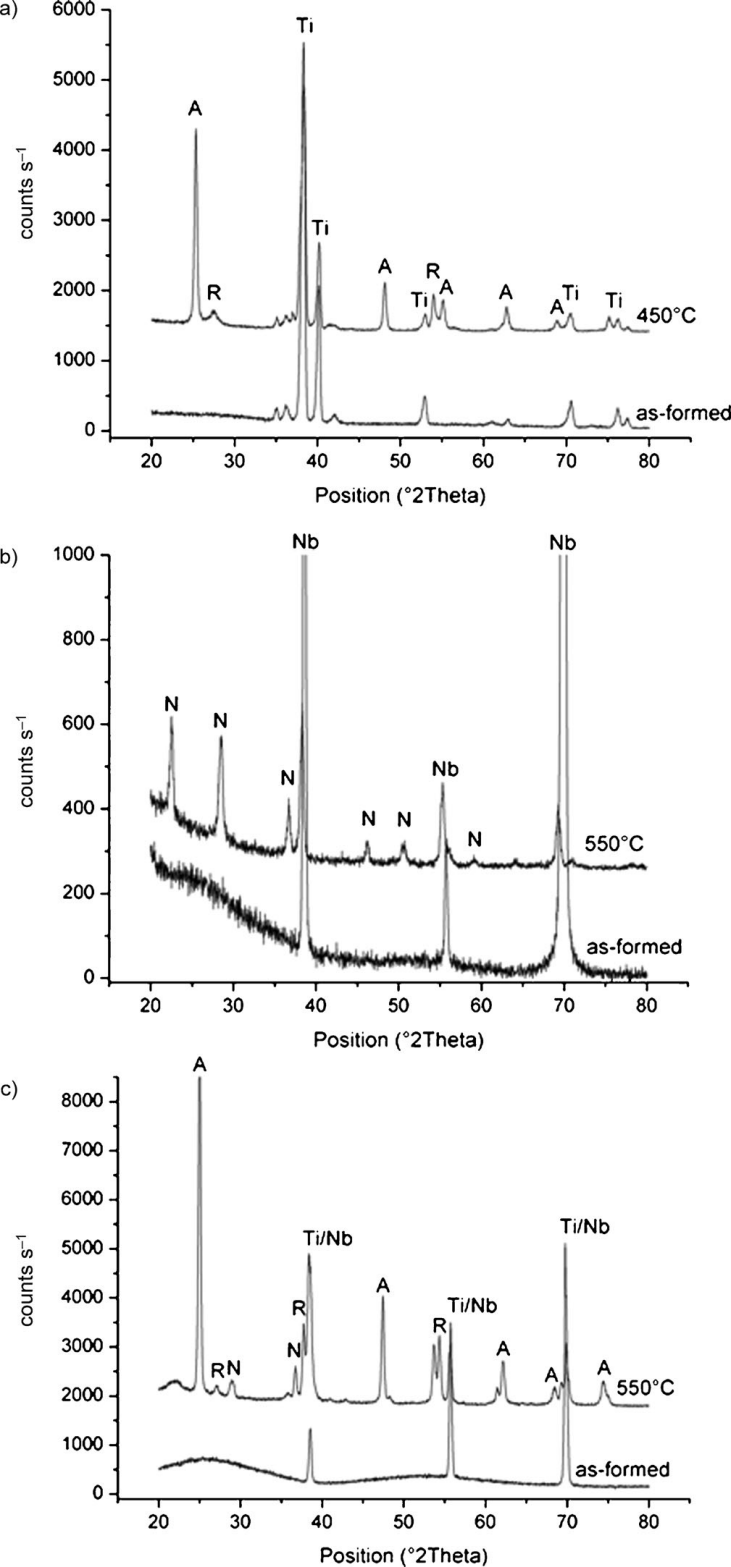
XRD pattern of the as-formed and annealed oxide layer: a) Ti anodized in 0.1 m HNO_3_, showing distinct anatase TiO_2_ peaks; b) Nb anodized in 0.2 m NH_4_NO_3_, resulting in the formation of Nb_2_O_5_; c) Ti45–Nb alloy anodized in 0.2 m NH_4_NO_3_, where a mixed oxide consisting of anatase TiO_2_ and Nb_2_O_5_ is observed. Anatase TiO_2_ (A); rutile TiO_2_ (R); Ti metal (Ti); Nb_2_O_5_ (N); Nb metal (Nb).

**Figure 4:**
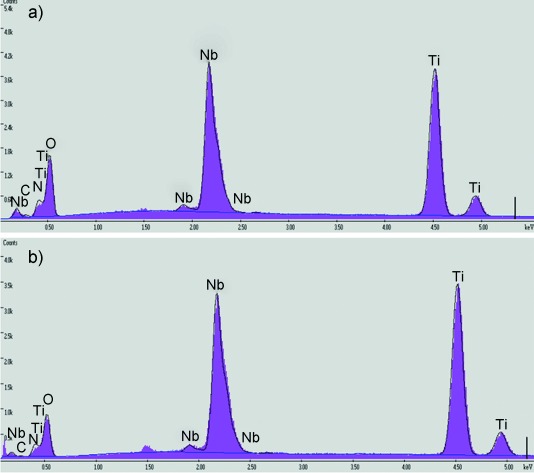
EDX spectra after annealing of the Ti45–Nb alloy grown in a) the nitric acid electrolyte, and b) the organic nitrate electrolyte.

Overall, the finding that nitrate-based electrolytes can be used to achieve self-organized oxide growth on metals with a very different electrochemical behavior, such as Ti and Nb (and their alloys), indicates that these electrolytes are very versatile. It is therefore likely that this approach can be applied to a wide range of metals to form ordered, through-hole morphologies, which might be, valuable as, for example, flow-through membranes or templates for the deposition of secondary materials.

The present work demonstrates that plain nitrate electrolytes can be successfully used to grow through-hole, self-organized oxide nanopore (or nanochannel) layers on Ti, Nb and their alloys. The observed pore diameter is in the range of 10–50 nm, and the layers typically have a thickness of 10 μm. Different optimized electrolyte conditions are needed to achieve successful growth of these structures, with key parameters being the water content and applied voltage. These findings suggest that, based on nitrate electrolytes, a wide range of metals can be anodized to form self-organized, through-hole porous oxide structures. Considering the plethora of applications of TiO_2_ and doped TiO_2_ nanoscale structures in particular, we believe that the present finding represents a novel platform for the fabrication of doped and well-defined metal oxide nanostructures.

## Experimental Section

Titanium and niobium foils (0.1 mm, 99.6 % Ti, 99.9 % Nb purity; Advent Materials, Oxford, UK) and a Ti–Nb alloy with a composition of 55 wt % Ti and 45 wt % Nb (ATI Wah Chang, Albany, USA) were used as substrates for anodization experiments. Prior to electrochemical treatments, the samples were degreased by sonication in acetone and ethanol, subsequently rinsed with deionized water and finally dried in a nitrogen stream. The samples were contacted with a copper plate and pressed against an o-ring in an electrochemical cell (1 cm^2^ exposed to the electrolyte). Anodization was carried out in 0.1 m HNO_3_ in a potential range of 10–40 V, or, alternatively, in ethylene glycol containing 0.2 m NH_4_NO_3_ with 3 % (*v*/*v*) H_2_O at potentials of 10–60 V. For the electrochemical experiments, a high-voltage potentiostat (Jaissle IMP 88) was used in a conventional three-electrode configuration with a platinum sheet as a counter electrode and a platinum wire as the pseudo reference electrode. All electrolytes were prepared from reagent grade chemicals.

A scanning electron microscope (Hitachi FE-SEM S4800) was employed for the morphological characterizations. The composition of the porous anodic oxide layers was investigated by energy dispersive X-ray (EDX) spectroscopy (Genesis system from EDAX). For structural characterization, X-ray diffraction (XRD) measurements were performed (X′Pert XRD system from Philips). Annealing of the samples was carried out in a furnace at 450 °C in air for Ti and at 550 °C in an N_2_ atmosphere for 1 h for Nb and the Ti45–Nb alloy.
